# Involving People with Lived Experience of Homelessness in Electronic Health Records Research

**DOI:** 10.23889/ijpds.v1i1.315

**Published:** 2017-04-19

**Authors:** Serena Luchenski, Sharon Clint, Rob Aldridge, Andrew Hayward, Nick Maguire, Alistair Story, Nigel Hewett

**Affiliations:** University College London; Groundswell; University of Southampton; Find & Treat; Pathway

## Objectives

We held a public engagement workshop to involve people with lived experiences of homelessness and social exclusion, experts by experience (EBE), in our research. The purpose of the workshop was to provide context to the findings of a review series on Inclusion Health and to inform future research using electronic health records (EHRs) of homeless and socially excluded people.

## Approach

Participants included 16 volunteers and one staff member from Groundswell (a registered charity that promotes inclusive solutions to homelessness), four academics, one clinical provider, and two note-takers/photographers. The full-day workshop was held at University College London. We used innovative participatory activities specifically designed for use with hidden and marginalised populations to stimulate open dialogue. Methods included general discussion, conceptual mapping, modelling, electronic voting, brainstorming, ranking, graphing, stakeholder analysis, and evaluation surveys. Activities were fast-paced and conducted in small groups, as the whole group, individually, or in pairs. The meaning of Inclusion Health, barriers that lead to exclusion, and values and actions that promote inclusion were discussed. Health statistics on homeless and other socially excluded groups were presented and informed conversations about EHR research, including data collection (new data versus collation of administrative data), data linkage, consent, anonymisation, data security, and `the surveillance society'.

## Results

Notes from the workshop and the evaluation surveys indicated that the workshop was engaging, inclusive, and successful. When clearly explained, EBE were positive towards research using EHRs to improve Inclusion Health, including data linkage of sensitive health and social data. EBE expressed that housing, advocacy, and psychosocial therapies were the most important interventions for improving health of people who are homeless; however, we found limited evidence for these interventions in our review. Overall, findings demonstrate the value of involving the people who have been socially excluded to develop research priorities and to interpret findings.

## Conclusions

Using a participatory and dynamic approach to involve people with lived experience of homelessness and exclusion is an effective public engagement methodology for complex topics such as EHR research and data linkage. Information provided in the workshop was useful for interpreting findings, identifying strengths and gaps in health and social services, and developing research and practice recommendations.

